# Nigral volume loss in prodromal, early, and moderate Parkinson’s disease

**DOI:** 10.1101/2023.08.19.23294281

**Published:** 2023-08-20

**Authors:** Jason Langley, Kristy S. Hwang, Daniel E. Huddleston, Xiaoping P. Hu

**Affiliations:** 1 Center for Advanced Neuroimaging, University of California Riverside, Riverside, CA, USA; 2 Department of Neurosciences, University of California San Diego, San Diego, CA, USA; 3 Department of Neurology, Emory University, Atlanta, GA, USA; 4 Department of Bioengineering, University of California Riverside, Riverside, CA, USA

**Keywords:** neuromelanin, substantia nigra, hyposmia, Parkinson’s disease

## Abstract

The loss of melanized neurons in the substantia nigra pars compacta (SNc) is a hallmark pathology in Parkinson’s disease (PD). Melanized neurons in SNc can be visualized *in vivo* using magnetization transfer (MT) effects. Nigral volume was extracted in data acquired with a MT-prepared gradient echo sequence in 33 controls, 83 non-manifest carriers (42 LRRK2 and 41 GBA nonmanifest carriers), 65 prodromal hyposmic participants, 105 *de novo* PD patients and 26 48-month PD patients from the Parkinson’s Progressive Markers Initiative. No difference in nigral volume was seen between controls and LRRK2 and GBA non-manifest carriers (*F*=0.076; *P*=0.927). A significant main effect in group was observed between controls, prodromal hyposmic participants, and overt PD patients (*F*=5.192; *P*=0.002). Longer disease duration significantly correlated with lower nigral volume (*r*=−0.252; *P*=0.010). This study shows that nigral depigmentation can be robustly detected in prodromal hyposmic participants and overt PD patients.

## 1. Introduction

Parkinson’s disease (PD) is a heterogeneous neurodegenerative disorder with a variety of motor and non-motor symptoms that can be clinically challenging to diagnose and manage, and there are currently no effective interventions to stop PD neurodegeneration. Empirical evidence suggests that PD-related neurodegeneration starts prior to symptom onset^1–5^ and understanding the magnitude and timing of PD-related neurodegeneration is essential to the development of early-stage diagnostic markers and outcome measures for clinical trials. At risk populations, such as patients with idiopathic rapid eye movement sleep behavior disorder (iRBD)^6^ or hyposmia with dopamine transporter deficits^7^, are ideal populations to examine neurodegeneration in the prodromal phase of PD since these patients are likely to phenoconvert to PD or other synucleinopathies^8–10^.

Neuromelanin loss in the substantia nigra pars compacta (SNc) is a hallmark pathology of PD^5,11,12^. The role of SNc in PD pathogenesis has been challenging to study *in vivo* due to a lack of tools to investigate PD-related nigral neurodegeneration in living patients. Recent work has found incidental magnetization transfer (MT) effects^13^ or explicit MT effects generated by MT preparation pulses^14–16^ can be used to generate contrast sensitive to neurons in SNc *in vivo.* Application of MT effects to image depigmentation has revealed PD-related reductions in MT contrast ratios in SNc^13,17–19^, nigral volume^14,20–25^, or the area of SNc in a single slice^26,27^. Nigral regions of interest, derived from images with MT effects, have also been used to examine PD-related microstructural changes^28,29^ or iron deposition^30,31^ in SNc.

In prodromal populations, much of the work using MRI has focused on developing diagnostic markers in populations with iRBD. Striatal segmentation in T_1_-weighted images revealed reductions in striatal volume in iRBD relative to controls^32^. Application of MT effects have found reduced locus coeruleus contrast^33^, SNc area^34^, and SNc volume^35^ in iRBD relative to controls. Hyposmic subjects with striatal dopamine transporter (123-I Ioflupane (DaTScan)) deficits have a high risk of phenoconverting^36^ and these results suggest that SNc may also be undergoing neurodegeneration in this population since DaTScan binding ratio is correlated with nigral volume^30^. However, the extent of SNc neuronal loss in hyposmic participants is unknown.

## 2. Methods

### 2.1 PPMI Overview

Data used in the preparation of this article were obtained from the Parkinson’s Progression Markers Initiative (PPMI) database (www.ppmi-info.org/data). For up-to-date information on the study, visit www.ppmi-info.org. Full inclusion and exclusion criteria for enrollment in PPMI can be found at www.ppmi-info.org. Institutional IRB approved the study for each site and subjects gave written informed consent.

### 2.2 Participants

Criteria for inclusion of subjects from the PPMI database used in this analysis were as follows: 1) participants must be scanned with a MT-prepared gradient recalled echo (GRE) sequence on a Siemens scanner. A total of 312 participants (33 controls, 42 LRRK2 NMC, 41 GBA NMC, 65 prodromal hyposmic participants, 105 *de novo* PD patients, and 26 PD patients at the 48-month time point) met these criteria. NMC of LRRK2 and GBA mutations were confirmed to have pathogenic variants of LRRK2 (G2019s) or GBA (N409S, R535H, L29Afs*18, L483P) genes. These participants were included in the analysis if they had UPDRS-III scores ≤ 5 at the 48-month time point and were not diagnosed with PD. Non-manifest LRRK2 and GBA participants were taken at the 48-month time point since that was the first time point containing MT-prepared GRE images. All prodromal participants used in the analysis had hyposmia based on the University of Pennsylvania Smell Identification Test (UPSIT), dopamine transporter deficits, and were not diagnosed with PD. The PD patients at the 48-month time point are not the same individuals from the *de novo* PD group. Imaging data were downloaded between July 2022 and January 2023.

### 2.3 MRI Acquisition

MRI data used in this analysis were acquired on Siemens MRI scanners. NM-MRI data were acquired using a 2D MT-prepared GRE sequence^15,16^: mean/min/max echo time (TE)=4.12 ms / 2.88 ms / 5 ms, mean/min/max repetition time (TR) = 478 ms / 450 ms / 691 ms, slice thickness 2 mm, in plane resolution 0.5×0.5 mm^2^, mean/min/max flip angle (FA) = 39.7°/22°/40°, mean/min/max bandwidth = 464 Hz/pixel / 122 Hz/pixel / 507 Hz/pixel, 16 contiguous slices, and magnetization transfer preparation pulse (300°, 1.2 kHz off resonance, 10 ms duration), 5 or 10 measurements. A T_1_ magnetization-prepared rapid gradient echo (MP-RAGE) sequence was acquired with the following parameters: TE/TR= 2.62 ms/2300 ms, inversion time = 900 ms, FA=9°, voxel size = 1.0 × 1.0 × 1.0 mm^3^ and used to derive a transform between Montreal Neurological Institute (MNI) common space and native T_1_-weighted images.

### 2.4 Image Processing

MRI data was processed using the FMRIB Software Library (FSL). A transformation was derived between each individual’s T_1_-weighted image and 2 mm Montreal Neurological Institute (MNI) T_1_-space using FMRIB’s Linear Image Registration Tool (FLIRT) and FMRIB’s Nonlinear Image Registration Tool (FNIRT) in the FSL software package using the following steps^37,38^. The T_1_-weighted image was brain extracted using the brain extraction tool (BET). Next, an affine transform was used to align the brain extracted T_1_-weighted images with the MNI brain extracted image. Finally, a nonlinear transformation was used to generate a transformation from individual T_1_-weighted images to T_1_-weighted MNI T_1_-space.

**Figure 1.**
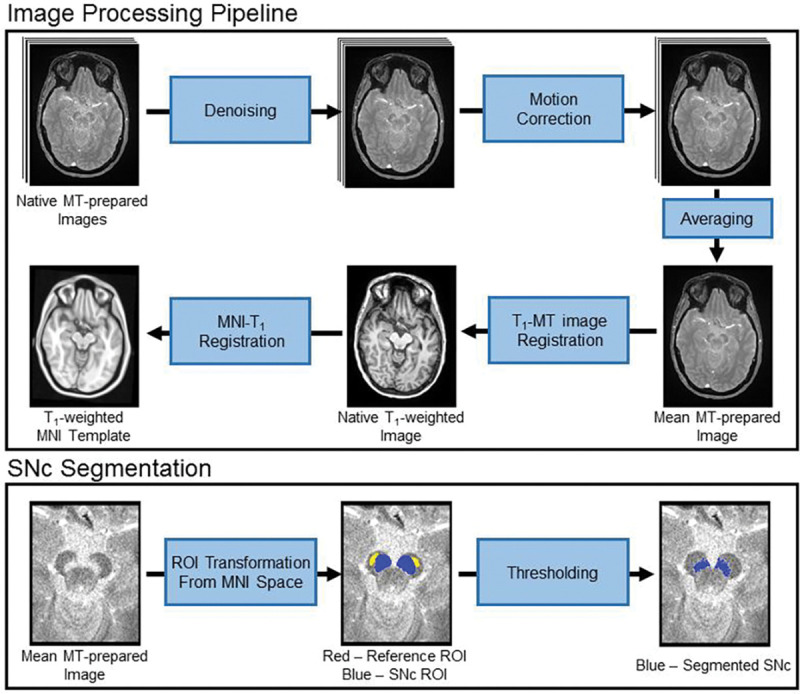
Schematics illustrating the processing steps for the MT-prepared GRE data and SNc segmentation procedure.

For each participant, individual MT-prepared GRE measurements were denoised^39^, corrected for motion by registering all the measurements to the first measurement using a rigid-body transform in FLIRT, and then averaged. Finally, a transform was derived between each individual’s T_1_-weighted image and the averaged MT-prepared GRE image with a boundary-based registration cost function. This transform was then inverted. This procedure is illustrated in Figure 1.

SNc volume was segmented in native space using an automated thresholding method. To ensure consistent placement of reference regions of interest (ROIs), a reference ROI in the cerebral peduncle was created using the MNI template and, for each subject, the cerebral peduncle ROI was transformed to individual MT-prepared GRE images using the MNI-T_1_ and T_1_-GRE transforms described in previous paragraphs. The transform was done in a single step to reduce interpolation. The use of standard space ROIs ensured that the reference ROI was placed in similar locations for each subject. The mean (denoted μ_ref_), and standard deviation (σ_ref_) of the signal intensities were measured in the reference ROI.

Next, a standard space SNc atlas was used to localize regions surrounding SNc for thresholding.^40^ This atlas was thresholded at a level of 5%, binarized, dilated, and transformed from standard space to individual MT-prepared gradient echo images. The ROIs for thresholding were dilated to ensure that the entire SNc was included for thresholding. Voxels in the resulting ROIs with intensity >μ_ref_+2.8σ_ref_ were considered to be part of SNc. This procedure is illustrated in Figure 1.

### 2.5 Statistical Analysis

All statistical analyses were performed using IBM SPSS Statistics software version 28 (IBM Corporation, Somers, NY, USA) and results are reported as mean ± standard deviation. A *P* value of 0.05 was considered significant for all statistical tests performed in this work. Normality of SNc volume was assessed using the Shapiro-Wilk test for each group and all data was found to be normal.

For demographic data, analysis of variance (ANOVA) was used to assess differences in age, years of education, UPDRS-III OFF score, and Montreal Cognitive Assessment (MoCA) of the prodromal PD, overt PD (*de novo*, moderate), and control groups. Chi square was used to examine differences in sex between groups.

The effect of genetic mutations (GBA, LRRK2) in NMC and controls on SNc volume was assessed with an analysis of covariance (ANCOVA) analysis controlling for age, total brain volume, MT-prepared GRE protocol used in the acquisition, and sex. The effect of group (control, prodromals, *de novo* PD, moderate PD) was tested with an ANCOVA for SNc volume controlling for sex, age, total brain volume, and the MT-prepared GRE protocol used in data acquisition. For all ANCOVAs, if the interaction was significant, post hoc comparisons between each pair of groups were performed using respective two-tailed t-tests.

The effect of nigral volume on clinical measures (MDS UPDRS-III OFF score, disease duration) was assessed by correlating nigral volume with clinical measures in the combined PD group (*de novo*+48-month). Correlations between clinical measures and nigral volume were performed using Pearson correlations in PD groups, controlling for age and total brain volume.

## 3. Results

### 3.1 Sample Demographics

No differences in sex (*Ps*>0.471), MoCA (*F*=0.823; *P*=0.442), or MDS UPDRS-III (*F*=0.220; *P*=0.802) score were observed between LRRK2 NMCs, GBA NMCs, and the control group without mutations. Significant differences were seen in age (*F*=4.386; *P*=0.015) and education (*F*=3.637; *P*=0.029) between LRRK2 NMCs, GBA NMCs, and the control group without mutations. Demographic information for this analysis is summarized in Table 1.

A significant difference in sex was observed between *de novo* PD and prodromal groups (*P*=0.018) but no difference was seen between the other groups (*P*s>0.202). A significant difference in age (*F*=5.259; *P*=0.002) was seen between the groups with the control group being younger, on average, as compared to the *de novo* PD (*P*<10^−3^) and 48-month PD (*P*=0.003) groups. The prodromal (*P*=0.017) and 48-month PD (*P*=0.049) groups were older, on average as compared to the *de novo* PD group. No difference in education (*F*=0.541; *P*=0.655) or MoCA (*F*=2.006; *P*=0.114) was seen between the groups. MDS UPDRS-III OFF score exhibited a significant group difference (*F*=108.619; *P*<10^−3^) with higher MDS UPDRS-III scores seen in the 48-month PD group relative to the prodromal, *de novo* PD, and control groups (*Ps*<10^−3^). Higher MDS UPDRS-III scores were seen in the *de novo* PD group as compared to the prodromal and control groups (*Ps*<10^−3^). The prodromal group exhibited higher MDS UPRDS-III scores relative to the control group (*P*<10^−3^). A significant difference was seen in UPSIT score between the groups (*F*=22.340; *P*<10^−3^) with the prodromal (*P*<10^−3^) and *de novo* PD (*P*<10^−3^) groups showing reduced olfactory function relative to controls. Demographic information for the control, prodromal, *de novo* PD, and 48-month PD groups is summarized in Table 2.

### 3.2 Non-manifest Comparisons

**Figure 2.**
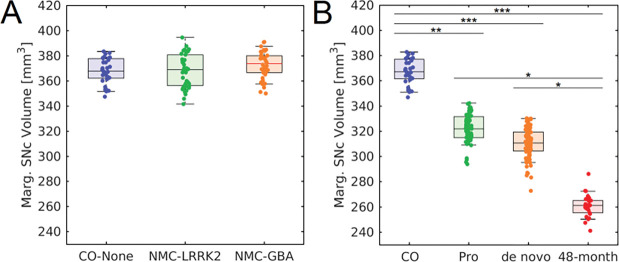
Comparisons of nigral volume marginal means and volume marginal means in LRRK2 NMC, GBA NMC, and non-carrier controls is shown in A. Comparisons of nigral volume marginal means in the control cohort, *de novo* PD cohort, and 48-month PD cohort is shown in B. *, **, and *** denote significant levels of *P*<0.05, *P*<0.01, and *P*<0.001, respectively.

**Figure 3.**
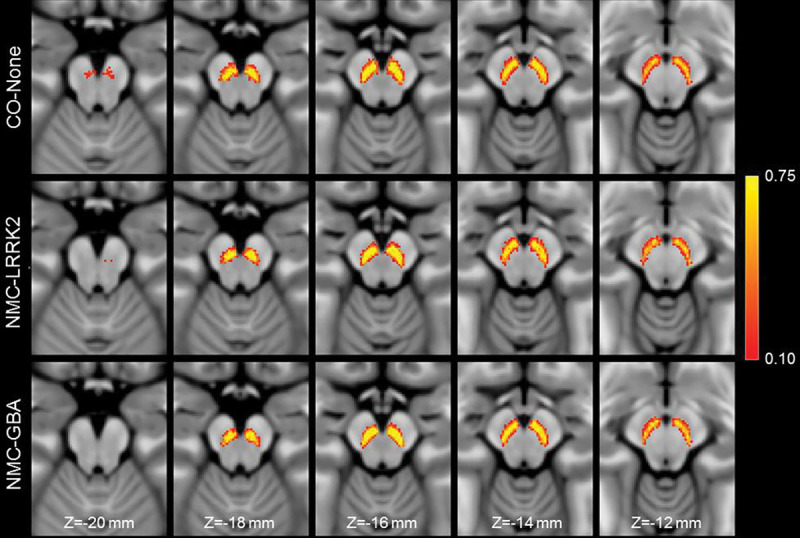
A comparison of SNc population mean volume in the control group (top row), non-manifest LRRK2 carriers (middle row), and non-manifest GBA carriers (bottom row). For each group, the SNc population mean volume was created by transforming SNc masks from individual participants to MNI space and then averaging.

The effect of LRRK2, GBA, and no genetic mutation on nigral volume in nonmanifest carriers and controls was tested with an ANCOVA analysis with the number of measurements in the acquisition protocol, total brain volume, sex, and age as covariates. ANCOVA analysis revealed no difference in nigral volume between NMCs of LRRK2 and GBA mutations and controls (*F*=0.076; *P*=0.927). The number of measurements in the acquisition protocol (*F*=0.209; *P*=0.648), total brain volume (*F*=3.304; *P*=0.072), and age (*F*=3.233; *P*=0.075) were not significant covariates in the model. These comparisons are shown in Figure 2. Sex was a significant covariate in the model (*F*=8.974; *P*=0.003). A spatial comparison of mean population SNc volume in the LRRK2 NMCs, GBA NMCs, and controls is shown in Figure 3.

### 3.3 Nigral Volume Group Comparisons

**Figure 4.**
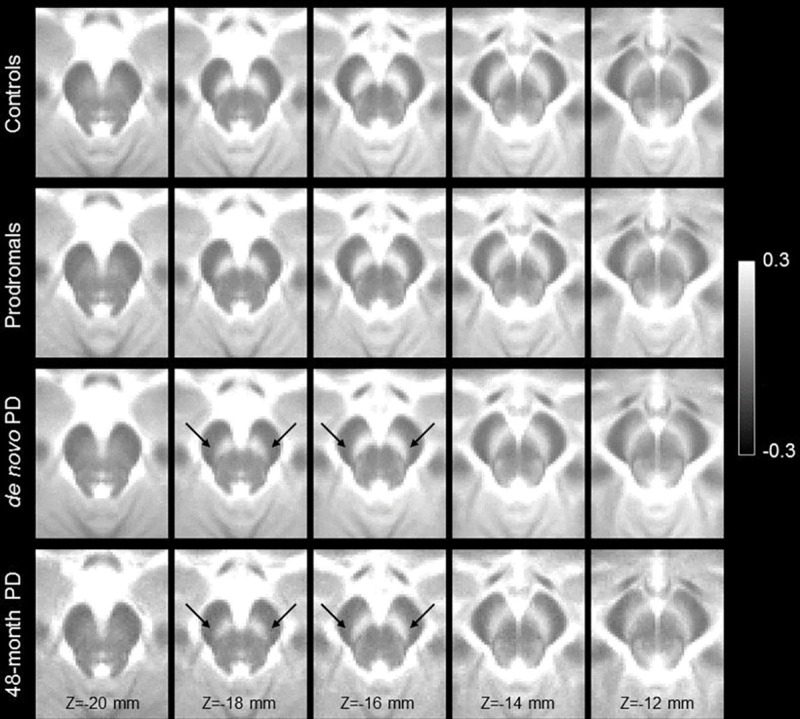
A comparison of mean SNc contrast in control (top row), prodromal (second row), *de novo* PD (third row), and 48-month PD (bottom row) groups. Reduced contrast can be seen in the prodromal and PD groups as compared to the controls in slices Z=−18 mm and Z=−16 mm. For each group, the mean MTC image was created by transforming MTC images from individual participants to MNI space and then averaging. Arrows point to the regions exhibiting a loss of contrast in the *de novo* PD and 48-month PD groups as compared to controls.

**Figure 5.**
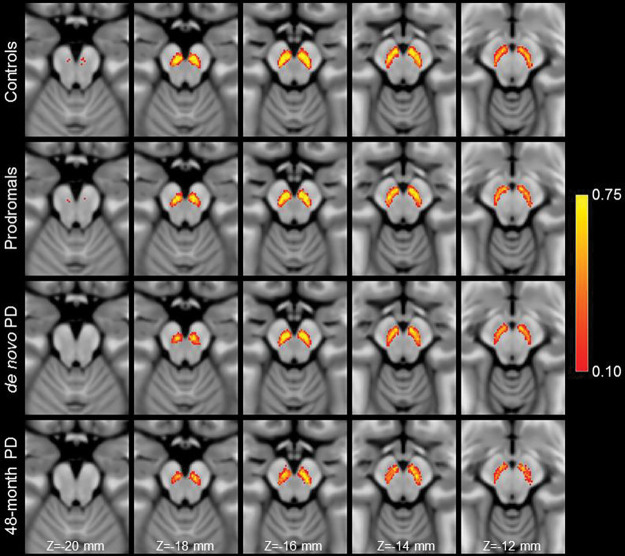
A comparison of SNc population means in control (top row), prodromal PD (second row), *de novo* PD (third row), and 48-month PD (bottom row) groups. Reduced volume can be seen in all pathologic groups as compared to the controls in slices Z=−18 mm and Z=−16 mm. For each group, the SNc population mean was created by transforming SNc masks from individual participants to MNI space and then averaging.

Figure 4 shows a comparison of mean MTC images in the control group (noncarriers), hyposmic prodromal group, *de novo* PD group, and 48-month PD group. The effect of group (control, prodromal, *de novo* PD, 48-month PD) on SNc volume was assessed using an ANCOVA with number of measurements in the NM protocol, age, total brain volume, and sex as covariates. A significant main effect of group (*F*=5.192; *P*=0.002) revealed reduced SNc volume in the prodromal (*P*=0.028), *de novo* PD (*P*=0.002), and 48-month PD (*P*<10^−3^) groups relative to the control group. 48-month PD group showed reduced nigral volume as compared to the prodromal (*P*=0.031), and *de novo* PD (*P*=0.049) groups. No significant difference was observed between *de novo* PD and prodromal groups (*P*=0.537). Sex was a significant covariate in the model (*F*=13.823; *P*<10^−3^) but NM protocol (*F*=0.830; *P*=0.363), age (*F*=0.003; *P*=0.957), and total brain volume (*F*=0.756; *P*=0.385) were not significant covariates. A comparison of SNc population mean volumes is shown in Figure 5 and marginal means for each group are summarized in Table 3.

The effect of genetic mutation (GBA, LRRK2) on SNc volume in the PD-group at the 48-month time point was tested using an ANCOVA with the number of measures in the NM protocol and sex as covariates. No main effect of group (*F*=0.822; *P*=0.496) was seen in SNc volume in the 48-month PD group (GBA: 259 mm^3^ ± 113 mm^3^; LRRK2: 262 mm^3^ ± 106 mm^3^). Sex (*F*=0.910; *P*=0.351) and NM protocol (*F*=1.624; *P*=0.641) were not significant covariates in the model. Reduced SNc volume was seen in PD patients at the 48-month time point with the LRRK2 mutation as compared to nonmanifest LRRK2 carriers (*F*=3.828; *P*=0.032). Similarly, reduced SNc volume was observed in PD patients at the 48-month time point with GBA mutation as compared to nonmanifest GBA carriers (*F*=6.113; *P*=0.005).

### 3.4 Clinical Correlations

SNpc volume differentiated SWEDD from PD better than SNpc The effect of disease severity (MDS UPDRS-III OFF score) and disease duration on nigral volume in the combined (*de novo*+48-month) PD group was assessed with Pearson correlations, controlling for age and brain volume. A significant correlation was seen between nigral volume and disease duration (*r*=−0.252; *P*=0.010) with longer disease duration correlated with lower nigral volume. No association was observed between nigral volume and MDS UPDRS-III OFF score (*r*=−0.048; *P*=0.640).

## 4. Discussion

This study examined PD-related SNc degeneration in prodromal hyposmic participants, *de novo* PD participants, and moderate PD participants. Standard space ROIs were used to calculate CNR and define SNc regions used in the thresholding-based segmentation procedure. This method has been shown to exhibit high scan-rescan reproducibility^41–43^. Application of the method found no difference in nigral volume between NMC of LRRK2 and GBA mutations and controls. Significant volume loss was seen in SNc of the prodromal PD group as well as in both PD groups as compared to controls. In addition, nigral volume in the moderate PD group (48-month PD) was reduced as compared to the *de novo* PD and prodromal groups. Further, SNc volume was similar for individuals with LRRK2 and GBA genetic mutations at the 48-month time point.

A prior study examining striatal binding ratio from dopamine transporter imaging (123-I Ioflupane DaTScan) in LRRK2 NMCs in the PPMI dataset found no difference in striatal binding ratio between LRRK2 NMCs and non-carrier controls at baseline and observed no progression in striatal binding ratio in follow-ups over two years^44^. As DaTScan striatal binding ratio is correlated with nigral volume^30^, these results suggest that LRRK2 NMCs will have similar nigral volume as controls. Our analysis revealed no difference in nigral between controls and LRRK2 NMCs at the 4-year time point. These results may be due to incomplete penetrance of the LRRK2 mutations as only 5 of 175 of the NMC LRRK2 participants in PPMI have converted to PD or maybe due to slower progression of LRRK2 NMCs as compared to other prodromal groups.

Olfactory dysfunction is a common symptom of PD^45^ and may precede clinical diagnosis by at least 4 years^46–48^. The prodromal hyposmic participants used here have dopamine transporter deficits and these participants are highly likely to phenoconvert to PD^36,49^. Dopamine transporter deficits suggest this population is experiencing nigral volume loss^30^. Consistent with this posit, reduced nigral volume was observed in the prodromal hyposmic group as compared to controls. Similar reductions in volume in the nigrostriatal system have been found in other PD prodromes likely to phenoconvert, such as iRBD^32,34,35^. Taken together, these results suggest the nigrostriatal system is undergoing neurodegeneration in the prodromal period of PD.

Reductions in contrast in MT-prepared GRE images were seen in the lateral and ventral portions of SNc in both PD groups as compared to controls (see Figure 2). Contrast in these regions is further reduced in the 48-month PD group as compared to the *de novo* PD group, suggesting that the depletion of melanized neurons continues as PD progresses. However, further longitudinal studies are needed to verify this observation. The loss of nigral volume result agrees with prior studies which found reductions in nigral width^27,50^, loss of contrast in the posterior portion of SNc^25^, or a loss of contrast in the lateral-ventral portions of SNc^19^. Further, these regions have been shown to overlap with nigrosome-1, the subregion of SNc with the greatest loss of melanized neurons^51,52^, and loss of contrast in these regions may be due to depletion of melanized neurons in nigrosome-1.

As compared to controls, reductions in contrast and volume were observed in the caudal and ventral portions of SNc in both PD groups with the 48-month PD group (see Figures 4 and 5). In particular, greater reductions in nigral contrast in these regions were seen in the Z=−18 mm and Z=−16 mm slices as compared the *de novo* PD group. Interestingly, when the PD groups were combined, a negative association was found between disease duration and nigral volume. These results suggests that loss of nigral neurons continues as PD progresses from the *de novo* stage into moderate stage of PD and agree with earlier studies that found nigral volume to be related to disease duration^23^. However, because this analysis employed a cross-sectional design with newly diagnosed and moderate PD individuals, a longitudinal study examining nigral volume and contrast in prodromal and *de novo* patients and following them over the course of PD is necessary to verify these findings.

There are several caveats to this study. First, the study employed a cross-sectional design to examine changes in nigral volume in prodromal, *de novo,* and moderate PD. A true longitudinal study is needed to fully assess longitudinal changes in nigral volume over the course of prodromal and overt PD. Second, in contrast to earlier studies^25^, no correlation was seen between nigral volume and MDS-UPDRS-III score. This lack of correlation may be due to a lack of PD patients with more severe motor symptoms. Third, results examining nigral volume at the 48-month time point in LRRK2 and GBA carriers should be interpreted with caution and larger multi-contrast imaging studies examining the effect of genotype on nigral characteristics in PD are needed. Finally, significant heterogeneity was seen in the scan parameters of the MT-prepared GRE sequence. Scan parameter was used as a control in the ANCOVA analysis to combat this heterogeneity and scan parameter was not found to be a significant contributor to the model.

The current findings provide additional evidence that MT-effects robustly detect nigral depigmentation in prodromal hyposmic, *de novo* PD, and moderate PD groups. The moderate (48-month time point) PD group experienced greater PD-related nigral volume loss as compared to the prodromal and *de novo* PD groups. Genetic mutation was not found to influence nigral volume in NMCs. Finally, protocol was not found to have a significant effect on nigral volume.

## Figures and Tables

**Table 1.** Demographic information for the analysis examining effect of genetic mutation on nigral volume in non-manifest subjects. Data is presented as mean ± standard deviation unless noted otherwise. ANOVAs were used for group comparisons of age, education, UPDRS-III, and MoCA from which *P* values are shown. MDS UPDRS-III - Movement Disorders Society Unified Parkinson’s Disease Rating Scale Part III; MoCA - Montreal Cognitive Assessment.

	CO (*N*=33)	Non-manifest Carriers		*P*
		LRRK2 (*N*=42)	GBA (*N*=41)	

Sex [M/F]	[19/14]	[20/22]	[17/24]	>0.471
Age [years]	59.9 ± 13.0	65.8 ± 7.3	64.6 ± 6.0	**0.015**
Education [years]	16.6 ± 3.8	18.0 ± 2.2	18.4 ± 2.5	**0.029**
MoCA	27.4 ± 2.1	27.9 ± 1.8	27.5 ± 1.9	0.442
MDS UPDRS-III	1.7 ± 2.0	1.6 ± 2.1	1.9 ± 1.8	0.802

**Table 2.** Demographic information for the groups used in the Control-PD pathology analysis. Data is presented as mean ° standard deviation unless noted otherwise. ANOVAs were used for group comparisons of age, education, UPDRS-III, and MoCA from which *P* values are shown. MDS UPDRS-III was measured in the OFF state. UPSIT scores were not released for the 48-month PD participants. MDS UPDRS-III - Movement Disorders Society Unified Parkinson’s Disease Rating Scale Part III; UPSIT - University of Pennsylvania Smell Identification Test; MoCA - Montreal Cognitive Assessment.

	CO (*N*=33)	Prodromal (*N*=65)	*de novo* PD (*N*=105)	48-month PD (*N*=26)	*P*

Sex [M/F]	[19/14]	[27/38]	[67/38]	[14/12]	**>0.018**
Age [years]	59.9 ± 13.0	67.3 ± 5.9	64.0 ± 9.8	67.8 ± 7.6	**0.002**
LRRK2/GBA/None	0/0/33	0/0/65	3/1/101	9/17/0	-
Disease Duration [years]	-	-	1.1 ± 0.9	6.7 ± 2.1	<**10**^**−3**^
MDS UPDRS-III OFF score	1.7 ± 2.0	5.9 ± 4.6	22.6 ± 9.7	28.9 ± 11.9	**<10** ^**−3**^
Hoehn & Yahr	0.0 ± 0.0	0.2 ± 0.5	1.7 ± 0.5	2.1 ± 0.4	**<10** ^**−3**^
UPSIT	33.1 ± 5.4	26.3 ±7.5	23.7 ± 7.7	-	**<10** ^**−3**^
Education [years]	16.6 ± 3.8	17.1 ± 2.4	16.6 ± 3.0	16.3 ± 3.6	0.655
MoCA	27.4 ± 2.1	26.3 ± 2.3	26.9 ± 2.5	26.1± 2.9	0.114

**Table 3.** Structure volumes from the marginal means in the control-pathology analysis. Data is presented as mean ± standard deviation. ANCOVAS were used for group comparisons of SNc volume from which the *P*-values and *F*-values are shown.

Brain Region	Control	Prodromal	*de novo* PD	48-month PD	*F*	*P*

SNc Volume	369.9 ± 108.7	322.0 ± 116.3	307.4 ± 112.3	260.9 ± 104.4	9.093	**<10^−3^**

## Data Availability

The data that support the findings of this study are available from the PPMI database (https://www.ppmi-info.org/access-data-specimens/data).

## References

[1] Dauer W. & Przedborski S. Parkinson’s disease: mechanisms and models. Neuron 39, 889–909 (2003). 12971891 10.1016/s0896-6273(03)00568-3

[2] Cheng H.C., Ulane C.M. & Burke R.E. Clinical progression in Parkinson disease and the neurobiology of axons. Ann Neurol 67, 715–725 (2010). 20517933 10.1002/ana.21995 PMC2918373

[3] Morrish P.K., Rakshi J.S., Bailey D.L., Sawle G.V. & Brooks D.J. Measuring the rate of progression and estimating the preclinical period of Parkinson’s disease with [18F]dopa PET. J Neurol Neurosurg Psychiatry 64, 314–319 (1998). 9527140 10.1136/jnnp.64.3.314 PMC2170010

[4] Tang C.C., Poston K.L., Dhawan V. & Eidelberg D. Abnormalities in metabolic network activity precede the onset of motor symptoms in Parkinson’s disease. J Neurosci 30, 1049–1056 (2010). 20089913 10.1523/JNEUROSCI.4188-09.2010 PMC2866050

[5] Fearnley J.M. & Lees A.J. Ageing and Parkinson’s disease: substantia nigra regional selectivity. Brain 114, 2283–2301 (1991). 1933245 10.1093/brain/114.5.2283

[6] Elliott J.E., Baseline characteristics of the North American prodromal Synucleinopathy cohort. Ann Clin Transl Neurol 10, 520–535 (2023). 36751940 10.1002/acn3.51738 PMC10109527

[7] Siderowf A., Impaired olfaction and other prodromal features in the Parkinson At-Risk Syndrome Study. Mov Disord 27, 406–412 (2012). 22237833 10.1002/mds.24892 PMC6342466

[8] Miglis M.G., Biomarkers of conversion to alpha-synucleinopathy in isolated rapid-eye-movement sleep behaviour disorder. The Lancet. Neurology 20, 671–684 (2021). 34302789 10.1016/S1474-4422(21)00176-9 PMC8600613

[9] Schenck C.H., Rapid eye movement sleep behavior disorder: devising controlled active treatment studies for symptomatic and neuroprotective therapy--a consensus statement from the International Rapid Eye Movement Sleep Behavior Disorder Study Group. Sleep medicine 14, 795–806 (2013). 23886593 10.1016/j.sleep.2013.02.016 PMC8783206

[10] Vaswani P.A., Serial olfactory testing for the diagnosis of prodromal Parkinson’s disease in the PARS study. Parkinsonism & related disorders 104, 15–20 (2022). 36194902 10.1016/j.parkreldis.2022.09.007

[11] Zarow C., Lyness S.A., Mortimer J.A. & Chui H.C. Neuronal Loss Is Greater in the Locus Coeruleus Than Nucleus Basalis and Substantia Nigra in Alzheimer and Parkinson Diseases. Arch Neurol 60, 337–341 (2003). 12633144 10.1001/archneur.60.3.337

[12] Chan-Palay V. Locus Coeruleus and Norepinephrine in Parkinson’s Disease. Psychiatry and Clinical Neurosciences 45, 519–521 (1991). 10.1111/j.1440-1819.1991.tb02540.x 1722263

[13] Sasaki M., Neuromelanin magnetic resonance imaging of locus ceruleus and substantia nigra in Parkinson’s disease. Neuroreport 17, 1215–1218 (2006). 16837857 10.1097/01.wnr.0000227984.84927.a7

[14] Ogisu K., 3D neuromelanin-sensitive magnetic resonance imaging with semi-automated volume measurement of the substantia nigra pars compacta for diagnosis of Parkinson’s disease. Neuroradiology 55, 719–724 (2013). 23525598 10.1007/s00234-013-1171-8

[15] Chen X., Simultaneous imaging of locus coeruleus and substantia nigra with a quantitative neuromelanin MRI approach. Magn Reson Imaging 32, 1301–1306 (2014). 25086330 10.1016/j.mri.2014.07.003

[16] Langley J., A multicontrast approach for comprehensive imaging of substantia nigra. Neuroimage 112, 7–13 (2015). 25731994 10.1016/j.neuroimage.2015.02.045 PMC4415274

[17] Ohtsuka C., Changes in substantia nigra and locus coeruleus in patients with early-stage Parkinson’s disease using neuromelanin-sensitive MR imaging. Neurosci Lett 541, 93–98 (2013). 23428505 10.1016/j.neulet.2013.02.012

[18] Matsuura K., Neuromelanin magnetic resonance imaging in Parkinson’s disease and multiple system atrophy. Eur Neurol 70, 70–77 (2013). 23796701 10.1159/000350291

[19] Huddleston D.E., In vivo detection of lateral-ventral tier nigral degeneration in Parkinson’s disease. Hum Brain Mapp 38, 2627–2634 (2017). 28240402 10.1002/hbm.23547 PMC5385149

[20] Castellanos G., Automated Neuromelanin Imaging as a Diagnostic Biomarker for Parkinson’s Disease. Mov Disord 30, 945–952 (2015). 25772492 10.1002/mds.26201

[21] Langley J., Hwang K.S., Hu X.P. & Huddleston D.E. Nigral volumetric and microstructural measures in individuals with scans without evidence of dopaminergic deficit. Front Neurosci 16, 1048945 (2022). 36507343 10.3389/fnins.2022.1048945 PMC9731284

[22] Hwang K.S., Langley J., Tripathi R., Hu X.P. & Huddleston D.E. In vivo detection of substantia nigra and locus coeruleus volume loss in Parkinson’s disease using neuromelanin-sensitive MRI: Replication in two cohorts. PLoS One 18, e0282684 (2023). 37053195 10.1371/journal.pone.0282684 PMC10101455

[23] Biondetti E., The spatiotemporal changes in dopamine, neuromelanin and iron characterizing Parkinson’s disease. Brain 144, 3114–3125 (2021). 33978742 10.1093/brain/awab191 PMC8634084

[24] Vitali P., Substantia Nigra Volumetry with 3-T MRI in De Novo and Advanced Parkinson Disease. Radiology 296, 401–410 (2020). 32544035 10.1148/radiol.2020191235

[25] Schwarz S.T., Xing Y., Tomar P., Bajaj N. & Auer D.P. In Vivo Assessment of Brainstem Depigmentation in Parkinson Disease: Potential as a Severity Marker for Multicenter Studies. Radiology, 160662 (2016). 10.1148/radiol.2016160662 27820685

[26] Schwarz S.T., T1-weighted MRI shows stage-dependent substantia nigra signal loss in Parkinson’s disease. Mov Disord 26, 1633–1638 (2011). 21491489 10.1002/mds.23722

[27] Reimao S., Substantia nigra neuromelanin magnetic resonance imaging in de novo Parkinson’s disease patients. Eur J Neurol 22, 540–546 (2015). 25534480 10.1111/ene.12613

[28] Pyatigorskaya N., Comparative Study of MRI Biomarkers in the Substantia Nigra to Discriminate Idiopathic Parkinson Disease. AJNR Am J Neuroradiol 39, 1460–1467 (2018). 29954816 10.3174/ajnr.A5702 PMC7410545

[29] Langley J., Diffusion tensor imaging of the substantia nigra in Parkinson’s disease revisited. Hum Brain Mapp 37, 2547–2556 (2016). 27029026 10.1002/hbm.23192 PMC4905784

[30] Isaias I.U., Neuromelanin Imaging and Dopaminergic Loss in Parkinson’s Disease. Front Aging Neurosci 8, 196 (2016). 27597825 10.3389/fnagi.2016.00196 PMC4992725

[31] Langley J., Reproducible detection of nigral iron deposition in 2 Parkinson’s disease cohorts. Mov Disord 34, 416–419 (2019). 30597635 10.1002/mds.27608 PMC6608731

[32] Rahayel S., Abnormal Gray Matter Shape, Thickness, and Volume in the Motor Cortico-Subcortical Loop in Idiopathic Rapid Eye Movement Sleep Behavior Disorder: Association with Clinical and Motor Features. Cereb Cortex 28, 658–671 (2018). 28591814 10.1093/cercor/bhx137

[33] Garcia-Lorenzo D., The coeruleus/subcoeruleus complex in rapid eye movement sleep behaviour disorders in Parkinson’s disease. Brain 136, 2120–2129 (2013). 23801736 10.1093/brain/awt152 PMC3692035

[34] Pyatigorskaya N., Magnetic Resonance Imaging Biomarkers to Assess Substantia Nigra Damage in Idiopathic Rapid Eye Movement Sleep Behavior Disorder. Sleep 40(2017). 10.1093/sleep/zsx149 28958075

[35] Takahashi H., Imaging of the nigrostriatal system for evaluating the preclinical phase of Parkinson’s disease development: the utility of neuromelanin, diffusion MRI, and DAT-SPECT. Br J Radiol 95, 20210837 (2022). 34808066 10.1259/bjr.20210837 PMC8822574

[36] Siderowf A., Clinical and Imaging Progression in the PARS Cohort: Long-Term Follow-up. Mov Disord 35, 1550–1557 (2020). 32657461 10.1002/mds.28139

[37] Smith S.M., Advances in functional and structural MR image analysis and implementation as FSL. NeuroImage 23 Suppl 1, S208–219 (2004). 15501092 10.1016/j.neuroimage.2004.07.051

[38] Woolrich M.W., Bayesian analysis of neuroimaging data in FSL. NeuroImage 45, S173–186 (2009). 19059349 10.1016/j.neuroimage.2008.10.055

[39] Veraart J., Fieremans E. & Novikov D.S. Diffusion MRI noise mapping using random matrix theory. Magn Reson Med 76, 1582–1593 (2016). 26599599 10.1002/mrm.26059 PMC4879661

[40] Langley J., Hussain S., Flores J.J., Bennett I.J. & Hu X. Characterization of age-related microstructural changes in locus coeruleus and substantia nigra pars compacta. Neurobiol Aging 87, 89–97 (2020). 31870645 10.1016/j.neurobiolaging.2019.11.016 PMC7064384

[41] Langley J., Huddleston D.E., Liu C.J. & Hu X. Reproducibility of locus coeruleus and substantia nigra imaging with neuromelanin sensitive MRI. MAGMA 30, 121–125 (2017). 27687624 10.1007/s10334-016-0590-z

[42] Wengler K., He X., Abi-Dargham A. & Horga G. Reproducibility assessment of neuromelanin-sensitive magnetic resonance imaging protocols for region-of-interest and voxelwise analyses. Neuroimage 208, 116457 (2020). 31841683 10.1016/j.neuroimage.2019.116457 PMC7118586

[43] van der Pluijm M., Reliability and Reproducibility of Neuromelanin-Sensitive Imaging of the Substantia Nigra: A Comparison of Three Different Sequences. J Magn Reson Imaging 53, 712–721 (2021). 33037730 10.1002/jmri.27384 PMC7891576

[44] Simuni T., Longitudinal clinical and biomarker characteristics of non-manifesting LRRK2 G2019S carriers in the PPMI cohort. NPJ Parkinsons Dis 8, 140 (2022). 36273008 10.1038/s41531-022-00404-w PMC9588016

[45] Stern M.B., Olfactory function in Parkinson’s disease subtypes. Neurology 44, 266–268 (1994). 8309571 10.1212/wnl.44.2.266

[46] Ponsen M.M., Idiopathic hyposmia as a preclinical sign of Parkinson’s disease. Ann Neurol 56, 173–181 (2004). 15293269 10.1002/ana.20160

[47] Haehner A., Olfactory loss may be a first sign of idiopathic Parkinson’s disease. Mov Disord 22, 839–842 (2007). 17357143 10.1002/mds.21413

[48] Ross G.W., Association of olfactory dysfunction with risk for future Parkinson’s disease. Ann Neurol 63, 167–173 (2008). 18067173 10.1002/ana.21291

[49] Jennings D., Conversion to Parkinson Disease in the PARS Hyposmic and Dopamine Transporter-Deficit Prodromal Cohort. JAMA Neurol 74, 933–940 (2017). 28595287 10.1001/jamaneurol.2017.0985 PMC5710321

[50] Reimao S., Substantia nigra neuromelanin-MR imaging differentiates essential tremor from Parkinson’s disease. Mov Disord 30, 953–959 (2015). 25758364 10.1002/mds.26182

[51] Damier P., Hirsch E.C., Agid Y. & Graybiel A.M. The substantia nigra of the human brain. II. Patterns of loss of dopamine-containing neurons in Parkinson’s disease. Brain 122 (Pt 8), 1437–1448 (1999). 10430830 10.1093/brain/122.8.1437

[52] Damier P., Hirsch E.C., Agid Y. & Graybiel A.M. The substantia nigra of the human brain. I. Nigrosomes and the nigral matrix, a compartmental organization based on calbindin D(28K) immunohistochemistry. Brain 122 (Pt 8), 1421–1436 (1999). 10430829 10.1093/brain/122.8.1421

